# Ulcerated Jejunal Lipoma Causing Gastrointestinal Bleeding: A Case Report

**DOI:** 10.7759/cureus.73446

**Published:** 2024-11-11

**Authors:** Maria L Almeida, Marcus Costa, Tatiana Bezerra Regueira, Tomazo Prince Franzini, Thiago F Secchi, Rafaela F Oliveira, Ana Laura o Ferreira, Isabella Carpanetti

**Affiliations:** 1 Endoscopy, Hospital Nove de Julho, São Paulo, BRA; 2 Gastrointestinal Endoscopy, Hospital Nove de Julho, São Paulo, BRA

**Keywords:** capsule endoscopy, case report, laparotomy, lipoma, small intestine

## Abstract

Lipomas of the small intestine are rare and generally asymptomatic. However, they can present with obstructive symptoms or gastrointestinal bleeding that is difficult to localize.

A 63-year-old female patient was admitted to the emergency department with a complaint of melena for four days. Endoscopy and colonoscopy were performed without any abnormalities. The patient showed no further externalization of symptoms and had a suspicion of intussusception on a CT scan. A capsule endoscopy was performed, revealing an elevated and ulcerated lesion in the proximal jejunum near the Treitz angle. An attempt at enteroscopy was made, but it was suspended due to gastric stasis. The patient developed obstructive symptoms and underwent exploratory laparotomy, which required enterectomy. The histopathological examination confirmed that the lesion was an ulcerated lipoma.

Lipomas of the small intestine are rare and challenging to diagnose. The use of capsule endoscopy and enteroscopy allows for the diagnosis of these lesions, greater accuracy in localization, and, in some cases, treatment.

## Introduction

Lipomas are benign tumors formed by fat cells. This is a rare condition in the small intestine, where they are more frequently found in the duodenum and jejunum and can be sessile or pedunculated [1]. Most lipomas of the digestive tract are asymptomatic, except when they exceed 4 cm in diameter, potentially causing clinical manifestations such as hemorrhage, ulceration, intussusception, or intestinal lumen obstruction [2,3]. The clinical presentation depends mainly on the location, size, and complications associated with the lipoma. The diagnosis of intestinal lipoma is usually made using imaging methods such as computed tomography (CT) or magnetic resonance imaging (MRI). On CT, intestinal lipomas usually appear as well-circumscribed masses with fat attenuation. Endoscopy may also be helpful in diagnosing duodenal lipomas, which may appear as yellowish submucosal growths on the duodenal mucosa. The treatment generally depends on the presence of associated symptoms and complications. Asymptomatic lipomas generally do not require intervention and can be monitored clinically. However, in complicated cases, such as intestinal obstruction, hemorrhage, or intussusception, surgical resection of the lipoma and sometimes the affected intestinal segment may be necessary [3].

## Case presentation

**Figure 1:**
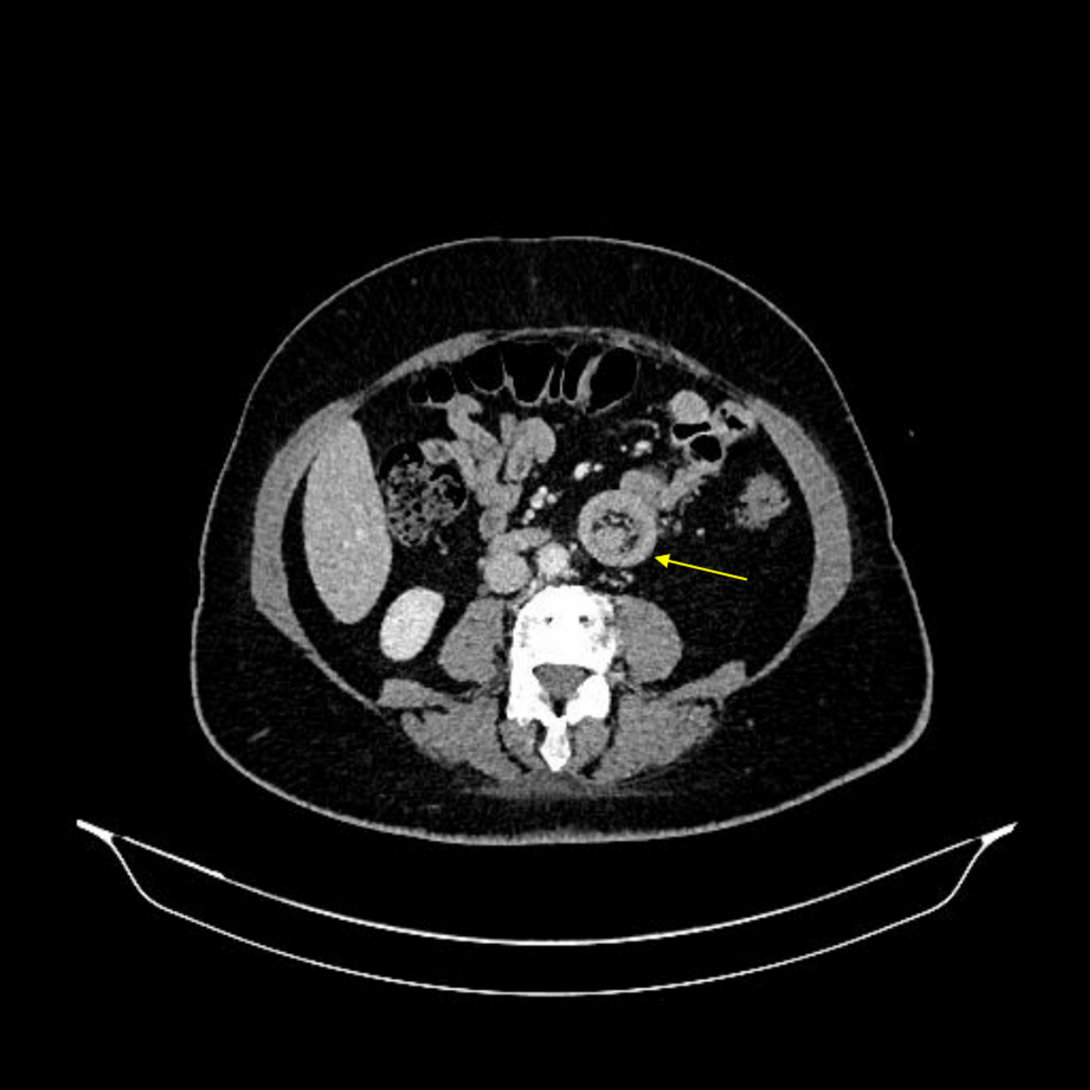
Abdominal CT scan image. The arrow indicates the location of the intussusception.

**Figure 2:**
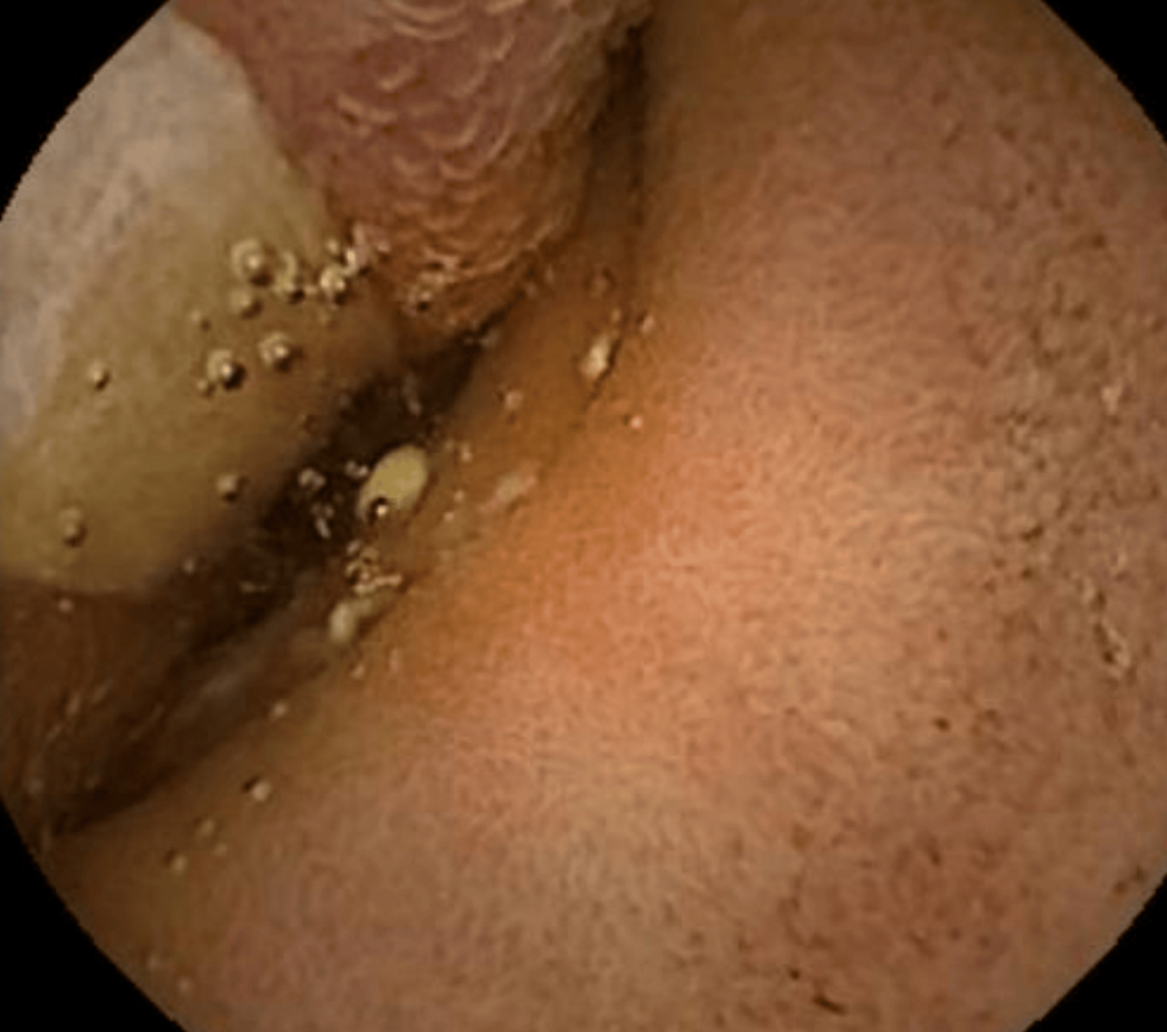
The endoscopic capsule image shows an ulcerated lesion with a central area covered by fibrin.

**Figure 3:**
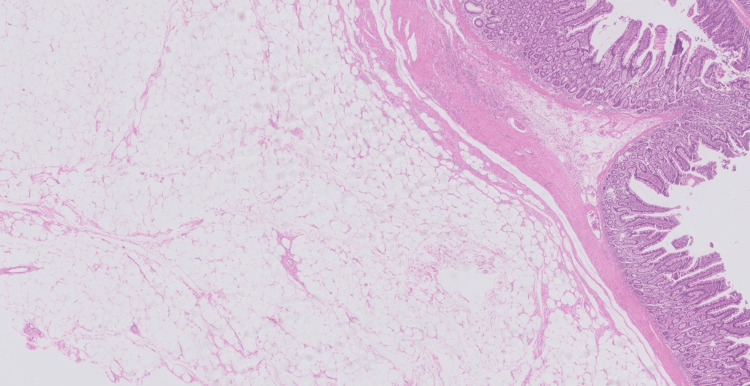
The anatomopathological examination confirmed the presence of yellowish cells and softened tissue compatible with lipoma.

A 63-year-old female patient presented with a history of melena for four days without any prior episodes, hematochezia, or weight loss. Her comorbidities included hypertension and overweight. She had a history of previous surgeries, including a cesarean section and umbilical hernia repair. Due to her stable clinical condition, the patient underwent an endoscopy, which revealed only small gastric polyps, and a colonoscopy, which showed a sigmoid colon polyp and a flat-elevated lesion in the transverse colon, both smaller than 10 mm, which were removed by polypectomy. Further investigation with an abdominal CT scan showed signs of jejuno-jejunal intestinal intussusception in the left flank, extending 8.5 cm, with a notable intraluminal lipomatous lesion of 4.0 cm at the head/distal portion of the intussusception. The lesion was likely an intestinal lipoma (Figure 1). The intussusception did not cause intestinal obstruction. The patient was then submitted to a high enteroscopy, but it was unsuccessful due to gastric stasis. A capsule endoscopy was subsequently performed, which identified an elevated and ulcerated lesion in the proximal jejunum segment (Figure 2). The patient later developed obstructive symptoms and underwent a laparoscopic approach, revealing adhesions from previous surgery. After these were released, the intestinal loops were examined from the Treitz ligament, and a 30 cm oval-shaped lesion was found in the intestinal lumen, causing upstream dilation and obstruction. A segmental enterectomy was performed, followed by enteroenteric anastomosis. The patient had a good clinical recovery with no complications and was discharged on the 10th postoperative day. The pathological examination of the resected small intestine segment measured 7.5 cm in length and 3.4 cm in diameter, with a smooth and shiny serosa. The mucosa appeared folded and brownish in color. A nodular formation measuring 3.5 × 2.5 cm was observed, consisting of yellow, soft tissue with the diagnosis of lipoma. The overlying mucosa was reactive with an area of ulceration and microvascular thrombi. Surgical margins were free of lesions. No signs of malignancy were observed (Figure 3).

## Discussion

Lipomas of the digestive tract are relatively rare benign tumors that primarily affect the colon (64%), small intestine (26%), duodenum (4%), stomach (3%), and esophagus (2%) [3]. When located in the duodenum, these lipomas predominantly occur in the second portion. Most intestinal lipomas are asymptomatic, but lesions larger than 4 cm can cause symptoms such as intussusception, obstruction, and bleeding [2]. These lesions typically originate in the submucosal layer of the intestine and grow toward the lumen, potentially developing a pedunculated configuration due to tumor growth and the traction forces exerted by the intestinal contents and peristaltic movements [3]. More than 200 cases of duodenal lipomas have been described, of which only 15 presented with severe upper gastrointestinal bleeding [4].

Characterization of these lesions through imaging methods (ultrasound and computed tomography) is often non-specific, and endoscopic biopsies tend to be negative due to the submucosal location. Thus, the definitive diagnosis generally depends on histological examination after surgical removal of the lesion. The differential diagnosis includes other benign tumors (such as adenomas of Brunner's glands, pancreatic adenomas, leiomyomas, angiomas, and aberrant pancreatic tissue) and malignant tumors (such as adenocarcinomas, lymphomas, carcinoid tumors, and leiomyosarcomas). While adenomas of Brunner's glands are more common in the first portion of the duodenum and primarily occur in the fifth and sixth decades of life, they are, like lipomas, generally asymptomatic findings [5].

The incidence of intestinal lipomas is higher in women, with a ratio of two women for every man [6]. The most common age for colon lipomas is between the fifth and sixth decades of life, while for the small intestine, it is between the sixth and seventh decades [7]. Lipomas in the small intestine are more frequent in the ileum, with the jejunum being less common, as identified in our case.

Surgical intervention is necessary in situations of obstruction, intussusception, perforation, or bleeding caused by intestinal lipomas. For lipomas larger than 2 cm, there is an increased risk of perforation, making the surgical approach preferable [7]. Some authors describe the endoscopic removal of lipomas up to 3.8 cm without complications, but, in general, surgical resection is indicated for larger lesions or those suspected of malignancy [8]. In the described case, surgical management was the most appropriate due to the development of acute obstructive abdominal syndrome.

## Conclusions

This article demonstrated a case of jejunal lipoma complicated with hemorrhage and intestinal obstruction. A rare clinical condition that must be taken into consideration as a differential diagnosis. Although rare, they should be remembered as a differential diagnosis. The implementation of minimally invasive exams, such as capsule endoscopy, allows identification and location with greater precision.

The prognosis for intestinal lipoma is generally excellent. This case highlights the importance of recognizing and appropriately managing jejunal lipomas. An early diagnostic and therapeutic approach is crucial to avoid unfavorable outcomes. After surgical resection, the majority of patients do not experience tumor recurrence, as does the patient in the clinical case who was followed up for six months after the surgical procedure and did not present any new findings or complications.

## References

[1] Tung CF, Chow WK, Peng YC, Chen GH, Yang DY, Kwan PC (2001). Bleeding duodenal lipoma successfully treated with endoscopic polypectomy. Gastrointest Endosc.

[2] Blanchet MC, Arnal E, Paparel P, Grima F, Voiglio EJ, Caillot JL (2001). Obstructive duodenal lipoma successfully treated by endoscopic polypectomy. Gastrointest Endosc.

[3] Geraci G, Pisello F, Arnone E, Sciuto A, Modica G, Sciumè C (2010). Endoscopic resection of a large colonic lipoma: case report and review of literature. Case Rep Gastroenterol.

[4] Weinberg T, Feldman M Sr (1955). Lipomas of the gastrointestinal tract. Am J Clin Pathol.

[5] Presti ME, Flynn MF, Schuval DM, Vollmar TM, Zotos VD (2015). Colonic lipoma with gastrointestinal bleeding and intussusception. ACG Case Rep J.

[6] Gao YP, Zhu JS, Zheng WJ (2004). Brunner's gland adenoma of duodenum: a case report and literature review. World J Gastroenterol.

[7] Virgilio E, Mercantini P, Cavallini M (2016). Is endoscopic resection a correct treatment for atypical gastrointestinal lipomas?. World J Clin Cases.

[8] Chehade HH, Zbibo RH, Nasreddine W, Abtar HK (2015). Large ileocecal submucosal lipoma presenting as hematochezia, a case report and review of literature. Int J Surg Case Rep.

